# Supplementary material for: A linear perception-action mapping accounts for response range-dependent biases in heading estimation from optic flow
Source: PLoS Comput Biol. 2025 Jun 9;21(6):e1013147. doi: 10.1371/journal.pcbi.1013147 (PMC12180628; doi:10.1371/journal.pcbi.1013147)
Supplement: Attachment — Submitted filename: ResponseLetter_20241103.docx [file pcbi.1013147.s008.docx]

**Response letter**

**(Comments in bold and purple)**

**Reviewer #1:**

**The current study examined heading perceptual bias reported in conflict in previous studies. The authors found that the conflict may be due to different range of perceptual report. Specifically, the bias is lager/overestimation when the scaled report range is large, and vice versa for the smaller bias/underestimation. The current study also excludes the motor report type, identifying the perceptual source. The finding is interesting.**

**Response:** We sincerely appreciate your thoughtful comments and suggestions.

**Major comment:**

**The result from the polar-report is robust and persuasive, yet if it is true, it may apply to the line-report. That is, when using different scaled range of the line, the over/under-estimation bias may also apply. It is worthwhile to test whether under longer lines, there would be overestimation and under shorter lines, there would be underestimation.**

**Response:** We thank the reviewer for this insightful suggestion. We have conducted an additional experiment to explicitly address this point. Specifically, we tested the effects of different line lengths (80, 111, and 142 visual degrees) on heading estimation biases. The results, consistent with our arc response findings, show that shorter lines (80°) led to underestimation while longer lines (111° and 142°) caused more overestimation. The results were shown in S3: Supplementary Figure 1. (The raw data have also been uploaded to OSF:

<https://osf.io/t47v6/?view_only=659d4bac8e4344f996eb8a51eb236d55>), which further support our model's predictions.

We also added the following paragraph to the result section: "*To further validate the influence of response range on heading estimation biases, we conducted an additional experiment in which participants reported their perceive heading by adjusting the mouse-controlled probe on a line of different lengths (e.g., 80°, 111°, and 142°). The results (Supplementary Fig. 1.) show that shorter lines led to underestimation while longer lines caused more overestimation, mirroring our findings with the arc responses. This highlights the generalization of the range effect in heading perception*." Please see Page 11, Lines 190-195.

**Minor comment:**

**1. Summary: "while these heading estimates are typically biased..." the sentence has grammar error.**

**Response:** We appreciate the reviewer's careful reading. We have corrected this sentence.

**2. Discussion: "estimates cam be solely..." typo error**

**Response:** We thank the reviewer for catching this typo. We have corrected it. Additionally, we have carefully reviewed the entire manuscript for other grammatical mistakes and have made the necessary corrections. Thank you very much!

**Reviewer #2:**

**This is an interesting study, in which the authors convincingly show that biases in heading perception (underestimation / overestimation in different studies) are dependent on the range of responses that participants were allowed to utilize when reporting their estimates.**

**Response:** We sincerely appreciate your thoughtful comments and suggestions.

**Major comments**

1. **The interpretation/ message inferred by ‘motor’, using terms like “motor mapping”, “motor stage” etc. seems imprecise (in relation the meaning of ‘motor’) and presumptuous. The authors themselves show that the results of these biases are independent of the motor action (mouse or keyboard control). So, clearly it is not motor per se. So what exactly is this mapping between? In fact, perhaps perception itself is affected by the range of options provided? And there are other candidate functions between perception and motor control (working memory?). I don’t see any evidence in the paper that justifies the assumption that this is motor mapping.**

**Response:** Regarding the imprecise use of motor-related terminology, we agree that our original language may have been misleading. To address this:

1. We have replaced some terms like "motor mapping" and "motor stage" with more neutral language such as "response mapping" or "perceptual-to-response transformation" throughout the manuscript.
2. We have added a discussion of potential cognitive processes involved in this mapping, acknowledging the possibility of perceptual effects and the involvement of other cognitive functions like working memory. We've included the following paragraph in the Discussion section: "*While our study demonstrates a robust transformation between the internal representation of heading direction and the reported estimate, we acknowledge that this process involves working memory and likely other processing stages. Thus, our results do not allow specific predictions about the specific nature of the sensorimotor transformation.*" Please see Page 22, Lines 363 to 366.
3. We have clarified that our model assumes the same percept across response conditions, but we now note that future studies could investigate whether the response range affects perception itself.

**2. The connection between the linear mapping offered here, and the model results (that show non-linear over/under-estimates) is not clear. How does linear mapping result in these non-linear results? I assume that this is probably due the prior – but this is not explained well enough. Especially when it comes the to the results/ figures.**

**3. Related to the above point - the deviations in the figures (that show under/over-estimation) are fit with linear functions, yet those deviations are not linear. Moreover, Fig. 2a – the four innermost headings (‘circle’ response) show the opposite effect to the four outermost headings on either side. This difference is seen for both constant speed and variable speed – so it seems robust (and a similar effect is seen in Fig 3b). This effect/difference is lost when fitting a straight line that is mostly constrained by the more peripheral headings. A linear fit also does not seem to work well for the ‘line’ response data. The use of a linear fit for behavior that systematically deviates from linear, seems questionable. Also, in Fig 3a,b there are systematic differences vs. linear. How are these reconciled with a linear mapping model?**

**Responses to 2 and 3:**

We appreciate the reviewer's observations regarding the linear fits and the nonlinear nature of our data. We agree that our original presentation may have been misleading with regard to the multiple use of ‘linear’.

The linear mapping in our model refers to the final step in our model inference process -- the transformation between the perceived heading direction and the reported estimate. However, the percept is the results of Bayesian inference, and thus inherently a non-linear process that combines the prior distribution of heading directions with the efficient coding of the sensory representation (i.e., the likelihood). The overall nonlinear response patterns emerge from the Bayesian observer model; the linear response mapping only scales the effects of these nonlinear components, leading to the observed patterns of over- and under-estimation that vary with response range.

Furthermore, the linear fits to the data (Figs. 2 and 3) only serve the purpose to quantify the empirical bias as over- (positive slope) or underestimation (negative slope). It is important to note that they do not represent fits of our model.

According to your comments 2 and 3, we therefore revised the manuscript as follows:

1. We have added a note in the caption of Fig. 2 to further explain that the linear fits are intended only to illustrate the general tendency towards over- or under-estimation, not to capture the full complexity of the data " … *To capture the general tendency towards over- or under-estimation, we fit a linear function to the measured bias data points (solid lines).*" Please see Page 8, Lines 138-140.

While we used linear fits to illustrate general trends, our Bayesian efficient coding model captures the full nonlinearity of the data. The model's ability to account for these complex patterns, including the differences between inner and outer headings, underscores its explanatory power and the necessity of considering nonlinear interactions in perceptual processes.

2. We have emphasized in the Results section that our Bayesian efficient coding model captures the nonlinear patterns in the data. “*In conclusion, our results demonstrate that an extended efficient Bayesian observer model based on a naturalistic prior on heading direction can well account for nonlinear patterns of participants heading estimation behavior (bias and standard deviation).*” Please see Pages 15-16, Lines 270-272.
3. While our model well accounts for the overall response pattern, it indeed does predict every fine detail of the data. In our revised manuscript we explicitly state this in the Discussion: “*While our model may not fully capture all aspects of participants reported heading perception, it provides valuable insights into the mechanisms underlying this effect.*” (Please see Page 23, Lines387 to 388.) However, we want to emphasize that we purposefully chose a relatively simple efficient Bayesian observer model (accounting for the non-linearities) because our focus was on the linear response-mapping relation between bias and variance on one hand, and the response range on the other.

**4. While the authors convincingly show that the range of possible responses is important, clearly there are also other factors at play for determining the bias (such as heading prior, as the authors show). So, statements like “entirely attributed” in the author’s summary, and “fully explained” in the Discussion etc. seem over the top.**

**Response:** We agree that our original phrasing may have overstated the role of response ranges. We have revised our language throughout the manuscript to more accurately reflect our findings. For example, in the Discussion, we now state: " *While our study demonstrates a robust transformation between the internal representation of heading direction and the reported estimate, we acknowledge that this process involves working memory and likely other processing stages. Thus, our results do not allow specific predictions about the specific nature of the sensorimotor transformation.* " Please see Page 22, Lines 363 to 366.

**Minor:**

**1. “two optic flow displays” is unclear, e.g., do the authors mean screens or stimuli?**

**Response:** We have revised this phrase to "two types of optic flow stimuli" to avoid confusion.

**2. ‘variable speed’ could be better defined. Sounds like speed fluctuates randomly during the stimulus. Although I eventually found the definition in the Methods, this could be better (at least generally, even without the specific details) explained earlier on in the text.**

**Response:** We have added a brief explanation when the term is first introduced: " *Note that variable speed in this these studies refers to a predetermined pattern of acceleration and deceleration during stimulus presentation”* More detailed parameters are provided in the Methods section. Please see Pages 4 and 5, Lines 81 to 82.

**3. 20-cm viewing distance seems quite close. With the need to focus/ fixate on the display center during the entire experiment, could this lead to fatigue (especially for those with corrected vision) or other issues?**

**Response:** We appreciate the reviewer's concern. The 20-cm viewing distance has been adopted in similar studies examining visual perception, particularly when a wide field of view is required. We carefully designed our experimental protocol to minimize fatigue, including 8 breaks to limit session durations. No participant reported any significant discomfort or fatigue during or after the experiments. It's worth noting that while our viewing distance is on the shorter end of the spectrum, it falls within the range used in previous studies, such as:

1. 20 cm (Sun, et al., 2020, Xu, et al., 2022)
2. 30 cm (Royden, Crowell, & Banks, 1994; Chen, DeAngelis, & Angelaki, 2013)
3. 43 cm (Warren & Saunders, 1995)
4. 45 cm (Warren, Jr., Morris, & Kalish, 1988)
5. 50 cm (Crowell & Banks, 1993)
6. 56.5 cm (Li, et al., 2015)
7. 65 cm (Edwards & Ibbotson, 2005)

**4. Some more explanations at other points in the paper can help. E.g., why does a cumulative distribution of the prior (Eq. 2) transform the heading direction from stimulus space into sensory space? The connection between the formula and its purported function are not explained.**

**Response:** We have added the following explanation to the Methods section: "*The cumulative prior distribution serves as a mapping function that transforms the physical stimulus space into a perceptual space. In this transformed space, the Fisher information becomes uniform, which, under the specific assumptions of our model, provides an efficient coding of sensory information. This approach has been widely adopted in perceptual modeling studies (e.g., Wei & Stocker, 2012, 2017; Ganguli D, Simoncelli EP. 2014; Polania et al 2019; Noel et al., 2021; Mao & Stocker, 2024; Hahn & Wei, 2024).*" Please see Page 27, lines 481 to 485.

**Reviewer #3:**

**The experiments are well-motivated and -conducted, and they may provide a plausible explanation for previously reported differences in heading biases across studies. However, I disagree with the strong conclusion expressed by the authors that these differences can be “fully explained by the influence of response range”. The authors do not address the fact that response ranges varied in previous studies because the stimulus ranges varied; the response range is typically expanded in proportion to the stimulus range. The same is not true in the present study; the stimulus range is held constant while the response range is manipulated. In this case, a response range that exceeds the stimulus range likely leads subjects to expand the range of responses they provide, as observed. This would not necessarily be the case if the stimulus range was similarly expanded. It is not clear that the current results would generalize to those more common conditions.**

**Response:** We sincerely appreciate the reviewer's thoughtful and constructive comments.

Our study presents a novel and comprehensive explanation for the contradictions in heading bias literature, supported by rigorous experimental data and a well-constrained computational model. While we acknowledge the reviewer's concerns, we respectfully assert that our model represents a significant advancement in correctly understanding psychophysical heading perception data.

(regarding response range influence) We acknowledge that our original conclusion might sound overstated. We have revised our language throughout the manuscript to more accurately reflect the scope of our findings.

We also added a paragraph in the Discussion section acknowledging the difference between our study design and previous studies: "*An important aspect of our experimental design is that we manipulated the response range while keeping the stimulus range constant. This differs from some of the previous studies where response ranges were typically expanded in proportion to the stimulus range. Future studies are necessary to more systematically explore how heading biases are affected by both stimulus and response ranges*" Please see Pages 21, Lines 358 to 362.

**Concerning the modeling, one prominent weakness is that the Bayesian prior is derived from psychophysical data and then this prior is used to predict the psychophysical data of the current study. Thus, the model in this case does not provide a strong test of the Bayesian model, i.e. that perception depends on the prior probability. It also means that the match between observed psychophysical performance and that predicted based on previous psychophysical performance is not surprising.**

**Response:** We appreciate the reviewer's observation regarding the potential circularity in our modeling approach. We have added the following paragraph in the discussion section to address this concern: “*We further tested our model using a neurally derived prior for heading direction. Specifically, we analyzed single cell recorded, neural activity in area MSTd of non-human primates in response to optical flow stimuli indicating heading direction [8]. Following a previous approach [19], we extracted Fisher information from the neural population responses in order to constrain the prior distribution. We found that this neurally derived prior for heading direction was similar to the prior from the psychophysical discrimination data, thus leading to similar modeling results. This alternative derivation of the prior further validated the robustness of our model and our general conclusions*” Please see Pages 19 and 20, Lines 313 to 320.

Additionally, we also want to clarify that our primary goal was not to test the validity of the Bayesian observer framework itself, but rather to examine how well such a perceptual model extended with a linear perception-response mapping could account for the range effects we (and previous studies) observed. By using a prior derived from previous data, we aimed to reduce free parameters and increase the model's predictive power across different experimental conditions.

**Another concern is the predictions of the model for backward heading directions. First, the prior derived from discrimination performance shows a peak for backward movements, but I am doubtful that straight backward heading occurs as frequently as this prior suggests. Second, the linear scaling of the response relative to the percept would predict that overestimation (or underestimation) will persist even for backward heading directions, but prior studies that have measured perception of backward heading do not observe this pattern.**

**Response:**
We thank the reviewer for highlighting this important point. While our current experiments focused on forward heading within a limited range, our model is capable of addressing backwards heading as well. Previous studies (Crane 2012, 2015; Cuturi 2013) have repeatedly shown that biases and variances in backwards heading responses are essentially symmetric in shape and magnitude to those for forward heading. Thus from a Bayesian perspective, this suggests that the prior as well as the likelihoods (stimulus uncertainties) around forward and backward directions are quite similar in magnitude and shape as well. Note also, that in natural conditions, humans frequently move their heads slightly backwards (and forwards) while being seated or standing, creating optical flow fields that are consistent with backward heading. Thus such a prior seems plausible.

We have extensively searched the literature for studies reporting response range-dependent over-and underestimation of backwards heading direction; unfortunately to no avail. Thus from our point of view, while forward and backward heading estimation seems to follow the same Bayesian inference process, it seems unclear whether the same range dependent bias patterns are present in studies of backward heading estimation. We thus have added the following discussion to address these concerns:

“*Although our current experiments focused on forward heading directions only, our model's predictions for backward heading perception are well-supported by existing literature. Previous studies investigating both forward and backward heading perception have shown bias patterns consistent with our model's predictions [6, 7, 36]. This suggests that our efficient Bayesian observer model and the assumed heading prior likely generalizes across the full range of heading directions. Future research, however, should explicitly validate that backward heading perception is similarly affected by the response range as forward directions, using experimental paradigms similar to those employed in our study. Additionally, exploring potential modifications to the model, such as incorporating categorical effects, could further enhance its ability to account for the full range of heading perception phenomena [30].*” Please see Page 22, Lines 366 to 376.

**Overall, the primary contribution of the work is the demonstration that response range impacts responses. The exploratory modeling is also interesting, but the only addition relative to previous models is the linear scaling of the response relative to the perceptual estimate. Most of the weaknesses outlined above are not satisfactorily addressed.**

**Response:**
We appreciate the reviewer's assessment of our work. While we agree that the demonstration of response range impact is a key contribution, we believe that our extended efficient Bayesian observer model provides valuable insights into the mechanisms underlying this effect. The linear scaling of the response relative to the perceptual estimate, while seemingly simple, offers a parsimonious explanation for a complex phenomenon and provides a framework for future investigations.

We have expanded our discussion of the model's implications and potential future directions to highlight the broader significance of our findings beyond the demonstration of the response range effect.

We appreciate the thoughtful comments, which has helped us refine our presentation, acknowledge limitations, and propose future research directions.
