# Supplementary material for: A linear perception-action mapping accounts for response range-dependent biases in heading estimation from optic flow
Source: PLoS Comput Biol. 2025 Jun 9;21(6):e1013147. doi: 10.1371/journal.pcbi.1013147 (PMC12180628; doi:10.1371/journal.pcbi.1013147)
Supplement: Attachment — Submitted filename: Rebuttal_Reviewers_Mar14.docx [file pcbi.1013147.s009.docx]

Saturday, March 15, 2025

Dear Editor, dear Reviewers.

Thank you for your thoughtful comments and suggestions. Based on your input, we have carefully revised our manuscript. Main changes include:

- an updated title: *A linear perception-action mapping accounts for response range-dependent biases in heading estimation from optic flow*;
- an extended discussion of the generalization of the model to other measured stimulus/response range conditions (i.e., 360 deg);
- an extended discussion of the nature of the inferred prior for heading direction. For the latter, we included a new Supplementary Figure showing the comparison of the assumed model prior based on measured discriminability threshold with an alternative estimate based on neural response characteristics in primate area MSTd;
- general wording changes to improve clarity and transparency of our presentation.

We think these changes have further improved the paper in terms of clarity of presentation and the implications of our results. Below, please find our point-by-point responses to your specific comments.

Sincerely,

Sun Qi, Linghao Xu, and Alan A. Stocker

**Point-by-point replies to comments (**Original comments are in purple).

**Editor:** I sympathize with the concerns of Reviewer 3. However, I believe there is also a lot of merit in the paper, both empirically, in eliminating alternative explanations for discrepancies across previous studies, and conceptually, in showing that a model that adds a simple linear scaling can alter the direction of biases, which does not seem obvious and so I wouldn't consider a trivial extension to existing models.

Response: Thank you. We think that our model in combination with its empirical validation provides a quantitative, interpretable, and principled explanation of seemingly conflicting perceptual behavior. To the best of our knowledge, no other model currently exists that provides a quantitative account for the influence of the response range on participants’ heading direction reports, or any other perceptual variables for that matter. Of course, we do not claim that our proposed model will be able to explain every kind of experimentally induced bias in perceptual reports. But we have clearly demonstrated that it does so for an extended set of empirical data while providing an intuitive understanding of a seemingly contradicting behavioral effect.

1) I think the reviewer's concerns can be adequately addressed as follows: 1) Address explicitly how the model predictions are affected when the response range imposed in the experiment is changed. As I understand the model, the experimentally imposed range of responses is irrelevant, and so you would predict no difference. It would be helpful to include a paragraph either in the results or discussion that addresses explicitly what the model predicts in this case.

Response: We thank the editor for providing these concrete instructions. Indeed, the original efficient Bayesian observer model predicts identical perceptual estimates regardless of response range because the response range is irrelevant for all current Bayesian observer models. A key contribution of our work is to demonstrate that response-range dependent biases can be parsimoniously explained by a simple post-perceptual transformation that maps the perceptual estimates to the reported responses. By doing so, we provide a unifying interpretation for a range of seemingly contrasting perceptual data showing over- and underestimation. The broader implication of our results is that the observed response-dependent biases are not perceptual.

In our revised manuscript, we have further emphasized this important aspect of our model in both the Results and Discussion sections. For example, we added the following paragraph to the Result section (Page 15, Lines 285-295): *“It is important to note that for the standard efficient Bayesian observer model [19] the experimentally imposed response range is irrelevant; it would predict identical perceptual biases regardless of how responses are collected. Our extended model builds on this well-proven theoretical framework and thus assumes that the underlying perceptual (Bayesian) processing remains constant across conditions. With the proposed linear mapping between participants’ percepts and their responses, it provides a parsimonious explanation for the observed systematic dependencies of participants’ heading reports on response range without the need for unrealistic assumptions of how the (invisible) response range could possibly directly affect the percept. As such, our results provide an intuitive, non-perceptual explanation for the apparently contrasting findings of both central (underestimation) and peripheral (overestimation) biases in heading estimation from optic flow.”*

2) Using an inferred prior is not unreasonable, but please check the paper carefully for how prior is described and possibly revise where necessary to clarify. For instance, the abstract suggests that the model assumes efficient coding "according to THE natural prior of freely behaving humans", which is misleading. It would be more accurate to say "according to A prior…”

**Response:** We agree that our writing was not precise in distinguishing between a directly measured statistical prior (which currently there are not reports in the literature about for heading direction) and an inferred prior (e.g., from psychophysical discrimination data).

In our revision, we are now very clear and unambiguous in that regard. For example, the revised Abstract explicitly states that the model uses (Page 15, Line 280) "*a prior inferred from heading discrimination data*" rather than "the natural prior”.

We also present an alternative estimate of the heading prior based on the neural response characteristics in the heading direction (optic flow) sensitive area of the primate visual system (area MSTd; Gu et al. 2010) and the assumption that these response characteristics are governed by efficient coding. This neural prior is qualitatively similar to the psychophysically estimated prior we used for our model (see new Supplementary Figure S2). Previous quantitative studies on orientation (Ganguli/Simoncelli 2010) and visual speed (Zhang/Stocker 2022) perception have shown that such neurally derived priors may represent good approximations of the statistical priors. While it is still an indirect measure of the statistical prior, it adds support to the notion that the choice of prior for our model may be a reasonable one.

3) Please respond to Reviewer 3's question about studies that have investigated 360° of heading stimuli.

**Response:** We now discuss the implications of having a 360 deg response range for the model and its predictions, as well as predictions for a 360 deg stimulus range; please, see our detailed response to Reviewer #3.

**Reviewer #1: My questions have been addressed well. I do not have further questions.**

**Response:** Thank you.

**Reviewer #2:** Overall the authors have updated the paper appropriately. Congrats on the well-done paper.

**Response:** Thank you.

I have one last minor comment: While the authors made changes in response to my first comment (about ‘motor’ mapping terminology) – the ‘motor mapping’ assumption is still quite evident in the paper, for example, in Figure 4b (and the Bayesian model description in general). IMHO, equating ‘motor’ with ‘action’ or ‘response’ (or everything that is not perception) is inaccurate and lacks nuance. Hence, I would encourage the authors to still further improve this aspect in their paper.

**Response:** We appreciate the reviewer's careful attention and thoughtful feedback. We agree that our previous use of "motor mapping" terminology might have lacked precision and potentially oversimplified the complex processes involved in transforming perceptual estimates into reported responses. With our revision, we have replaced any mentioning of “motor mapping” with “response mapping” or more general “perception-action transformation”. We hope this more general terminology reduces any potential misunderstanding and ambiguity.

**Reviewer #3:**

I thank the authors for responding to my comments. Unfortunately, I still maintain that this is a rather modest and incremental advance in our understanding, specifically the unsurprising observation that observers expand their responses to fill the provided responses range. This alone is not an impactful finding. If the study was able conclusively explain previously observed differences in heading bias reported across studies, this would add to the impact. However, the failure to manipulate stimulus range in proportion to response range does not allow reaching this conclusion. The insistence of the authors that they have conclusively demonstrated this is a significant weakness.

**Response:** We appreciate the reviewer's perspective on the scope and impact of our findings. However, we must respectfully disagree as we believe our study makes several novel and valuable contributions:

1. Foremost, our study indeed provides a unifying and coherent explanation for a wide range of results from previous studies that found divergent biases in participants’ heading estimates. It shows that the differences are neither caused by the specific response method (keyboard or mouse, line or arc), nor by the motion profile (acceleration/deceleration) of the optic flow patterns; see also our replication of the main experiments with line responses (Supplementary Fig.1). Rather, it demonstrates that the divergent bias patterns (and the variance in participants’ estimates) can be precisely pinpoint to the differences in response range of these studies, which we more systematically replicated characterized with our experiments.
2. We also introduce a model that provides a formal and quantitative testable description of our conceptually novel idea that the response range itself leads to a linear scaling of the perceptually inferred heading direction. To the best of our knowledge, our study is the first to address these contrasting bias effects with a quantitative model. We validate the power of the model by fitting it to data of the combined subject as well as individual subjects.
3. Even if one may disagree with the conclusion and implications of our study, our model nevertheless represents a precisely formulated hypothesis that can be quantitatively tested in future investigations. For example, if new evidence for a statistically defined prior becomes available, updated quantitative predictions for bias and variance in participants heading estimates can be immediately generated. As such our model offers the means for the constructive, iterative scientific process of hypothesis generation and empirical validation. We thus consider our work a quite valuable, scientific contribution, irrespective of whether the model ultimately needs to be modified or not.

Note that the statement that “observers … expand their responses to fill the provided response range” is not correct. In fact, the perception-action scaling is typically smaller than one, suggesting that observers typically compress their responses, and they only provide a response that matches their percept when being presented with an unrestricted response range. Thus, we don’t consider this empirical outcome trivial.

In response to the reviewer’s concern, we have extensively searched the literature for additional relevant datasets. However, we did not find datasets that contradict our results, nor datasets that independently manipulated both response and stimulus range. There are datasets that measured heading direction over 360 deg, though, which we address below.

We acknowledge that for our study we kept the stimulus range constant while manipulating the response range. This was a deliberate choice to isolate the effect of response range from other potential causes (e.g. response method, mouse vs keyboard). As such, of course, we do not strictly demonstrate that our findings and conclusions generalize beyond the experimental conditions tested. In order to be precise, we now explicitly state this in the Discussion (Page 22, Lines 381-391): *“Another important aspect of our experimental design is that we manipulated the response range while keeping the stimulus range constant. Future studies are necessary to more systematically explore how our results generalize to larger ranges of heading directions. This will also reveal whether the linear response mapping of our model extends uniformly across all heading directions or shows deviations at ecologically significant angles (such as straight ahead, sideways, or backward motion). This consideration of broader heading ranges also constrains our current version of the model. While we considered the scaling factor α a free model parameter, we note that for cases where both stimulus and response ranges span 360°, α must be constrained to equal 1 to avoid impossible predictions at extreme angles. Interestingly, with our unconstrained model fit, α is indeed approximately 1 for the largest response range. Thus, imposing such a constraint would not significantly affect our results.”*

In response to my three main criticisms, the authors have added to the text. While these additions acknowledge the criticisms, they do not alleviate them.

The first main criticism is covered above. To convincingly address this criticism, it would be necessary to collect additional data in which stimulus range is also varied. The addition of a few sentences acknowledging this weakness in the discussion is not sufficient.

**Response:** We understand the reviewer’s interest in data of experiments that systematically vary both. To do this properly, however, would require a series of experiments that parametrically and independently vary both response range and stimulus range. We think that such an additional, extensive empirical validation is beyond the scope of our study with its already extensive experimental and modeling components.

The second main criticism is the circularity of the model; deriving the prior from psychophysical data, then using that prior to predict psychophysical data. The authors have added a prior derived from neural data, however, the fact that neural and psychophysical data agree in this way has been previously reported, and once again, the prior is not derived from behavioral heading measures.

**Response:** We acknowledge the reviewer's concern. However, we do not agree that there is a problem of circularity. While we derive the prior from discrimination thresholds (measured in a different experiment from a different lab), we use it to explain a qualitatively quite different behavioral phenomenon - the systematic effect of response range on heading estimation biases. This represents a novel, model-based prediction rather than circular reasoning.

In our revision, we now more explicitly state that the prior used for our model predictions is an estimate and may not necessarily reflect the statistical prior of heading direction. However, we now also include a neurally derived prior for heading direction, extracted from an analysis of the population response characteristics of neurons in area MSTd of non-human primates [Gu et al., 2010]. Again, while not providing direct evidence, the qualitative convergence between the psychophysically and physiologically derived priors (see Supplementary Figure S2), and the fact that previous studies demonstrated instances where such neural priors indeed reflect the statistical prior (e.g., visual orientation, see Ganguli/Simoncelli 2010, Wei/Stocker 2012) provides further support for our choice of prior. Future research is necessary to provide a direct estimate of the statistical prior by carefully measuring the statistics of heading direction movements at the level of the retina.

The third main criticism is that the model requires a prior that is peaked for backward heading directions. Given that human locomotion is almost always in the forward direction, such a prior is unlikely. I am unconvinced by the authors’ rebuttal of this point.

**Response:** We understand the reviewer’s concern and agree that humans’ locomotion is typically forward. However, please note that heading direction movement is not limited to actual full body locomotion. Rather small heading movements already create strong optic flow signals that are exploited by the visual system to control head movements and stabilize head posture. These small head movements, on average, necessarily need to be balanced between forward and backward motion. Thus, it seems plausible that the prior may be more symmetric than expected based on full body locomotion behavior alone. However, as mentioned above, only direct statistical measurements at the level of the retina can determine the precise nature of the heading direction prior.

A related observation that is dismissed by the authors is that a linear transformation of responses has difficulties for heading directions beyond +/- 90 degrees. At this point, a bias towards “overestimation” of forward heading angles will become a bias toward “underestimation” of backward heading angles. In other words, the linear mapping cannot account for biases observed in studies that have investigated 360 deg of heading stimuli. In these studies, there is front-back symmetrical bias towards lateral heading directions. As I understand it, a simple linear response mapping cannot explain this front-back symmetry.

**Response:** We appreciate that the reviewer raises this important point. So far, we have not considered the implications of our model assumptions for larger stimulus ranges. A few previous studies have measured heading estimates from optic flow for the full 360 deg stimulus range (e.g., Crane 2012, Cuturi & MacNeilage, 2013). Obviously, for these experiments the response range needed also to be 360 deg. As the reviewer correctly points out, under these conditions, humans show a strikingly symmetric bias patterns for forward and backward heading (e.g., Fig.3B in Cutur/MacNeilage, 2013). However, it is important to note that our model can indeed account for these symmetric bias patterns for the full range if the scaling factor $\alpha$ is equal to 1. As we pointed out, our results show that participants do not expand their reports to fill out the response range, but rather increasingly compress their responses as a results of an increasingly constrained response ranges. Thus, this constraint naturally follows from our findings.

We run a simulation of the model to demonstrated this (prior as assumed in the manuscript, noise parameter as for the joint fit to Exp. 3; see Fig. 3). The model’s predicted bias pattern is shown below (Fig. R1) and clearly demonstrates that it accurately preserves the forward/backward symmetry in estimation bias under this condition.

Fig R1.

Because our study deliberately focused only on manipulating response range while keeping the stimulus range constant (±33°) we let the scaling factor $\alpha$ of our model to be a free parameter. However, it is important to note that for the largest response range (240 deg) the fit value for alpha is very close to 1 (1.05). Thus, imposing the additional constraint $\alpha$ ≤1 does not significantly affect our model fits, but allows the model to account for responses ranges up to the full circle.

Figure R1: Model predicted heading bias for 360 deg stimulus (and response) range with $\alpha$ =1.

We thank the reviewer to bring up this point. In our revision we have now added a new paragraph in the Discussion that addresses both the potential special status of sideways movements and the theoretical constraints on our model when extended to full 360° ranges. The paragraph clarifies that the extension to broader heading ranges raises interesting theoretical considerations that constrain our model and suggest directions for future research (Page 22, Lines 381-391):

*“Another important aspect of our experimental design is that we manipulated the response range while keeping the stimulus range constant. Future studies are necessary to more systematically explore how our results generalize to larger ranges of heading directions. This will also reveal whether the linear response mapping of our model extends uniformly across all heading directions or shows deviations at ecologically significant angles (such as straight ahead, sideways, or backward motion). This consideration of broader heading ranges also constrains our current version of the model. While we considered the scaling factor α a free model parameter, we note that for cases where both stimulus and response ranges span 360°, α must be constrained to equal 1 to avoid impossible predictions at extreme angles. Interestingly, with our unconstrained model fit, α is indeed approximately 1 for the largest response range. Thus, imposing such a constraint would not significantly affect our results.”*
