# Supplementary material for: A linear perception-action mapping accounts for response range-dependent biases in heading estimation from optic flow
Source: PLoS Comput Biol. 2025 Jun 9;21(6):e1013147. doi: 10.1371/journal.pcbi.1013147 (PMC12180628; doi:10.1371/journal.pcbi.1013147)
Supplement: Attachment — Submitted filename: Rebuttal_Reviewers_April28.docx [file pcbi.1013147.s010.docx]

**Point-by-point replies to comments (**Original comments are in purple).

**Reviewer #3:**

I thank the authors for addressing my previous comments. I maintain that additional revisions are needed to bring the claims in line with what the work has actually demonstrated.

My first main criticism was that the manuscript falsely claims that the current findings and model account for previously observed discrepancies in the heading perception literature, namely that different patterns of bias have been reported across studies.
These claims can still be found in the abstract, author summary, and main manuscript. I assert that this is a false claim because the current study only manipulates response range (not stimulus range). In contrast, in all previous studies in the literature, when response range was varied, stimulus range was also varied.
In order to demonstrate that a simple linear scaling of the response can account for different bias patterns in the literature, it would be necessary to vary both stimulus and response range, as has been done in previous studies**.** In contrast, in the current study, stimulus range was held constant and only response range was varied. Therefore, it is misleading to claim that the current results and modeling can conclusively account for previously observed discrepancies in reported heading biases. In fact the authors do concede this point in their response to reviewers: “As such, of course, we do not strictly demonstrate that our findings and conclusions generalize beyond the experimental conditions tested.”
Given this concession, it would seem necessary to remove all claims in the manuscript that the current model and results can conclusively explain previously observed discrepancies in the literature. Instead, the authors introduce a novel manipulation (of response range), observe novel effects of the manipulation (heading biases), and conceive a model to explain these effects. It would therefore seem appropriate to edit the abstract, author summary, and manuscript to more accurately reflect this specific contribution without making broader, unsupported claims about how the work generalizes.

**Response:**

In response to the reviewer’s remaining concerns and with the editor’s guidance, we have further revised our entire manuscript (Abstract, Author Summary, Introduction, and Discussion) to ensure our claims accurately reflect the specific contributions and limitations of our study. Specifically we have further moderated our language when discussing the relationship between our findings and the broader literature on heading biases. We have incorporated qualifiers such as *"potentially," "may," and "could"* where possible to reflect that while our findings offer a compelling model, further work exploring the full parameter space would be beneficial. We believe that this final revision satisfyingly addresses the reviewer's continued request for a more nuanced presentation of our conclusions regarding the generalization of our findings.

My second main criticism, namely that the prior is circular has been sufficiently acknowledged and addressed.
**Response:** Thank you.

My third main criticism has also been addressed. However, some additional commentary on this point may be warranted. In an additional paragraph in the discussion, the authors acknowledge that 360 stimulus range with 360 response range dictates that the linear scaling factor must be set to a value of 1. What they do not explicitly acknowledge is that setting this parameter equal to 1 is the same as eliminating their novel response mapping stage and reverting to pre-existing modeling approaches. Extending this reasoning, and in the spirit of parsimony, one might argue that such 360 studies are therefore preferable because they allow models with fewer parameters and also allow assessing sensory-perceptual processes while reducing (or perhaps minimizing) the risk of introducing response-scaling biases.

**Response:**

We thank the reviewer again for raising the issue of the 360 stimulus/response range during the review process. We also appreciate that the reviewer considers the concern now to be fully addressed.

However, we must respectfully disagree with the notion that the novelty of our results are diminished because of the constraint α=1 for a 360 stimulus range. Our empirical findings robustly demonstrate that the response range can exert a significant influence on reported heading estimates, mediating a systematic shift from underestimation biases with narrow ranges to overestimation biases with wider ranges, as well as a standard deviation that scales up with range. Furthermore, we demonstrated that a standard efficient Bayesian observer models, which implicitly assumes α=1, cannot explain the empirically measured range-dependent effect. We introduce and extended model that explicitly incorporates the scaling factor α as a parameter that varies monotonically with the response range. Consequently, models tailored exclusively for the 360/360 scenario represent merely a specific model instance (α=1) within the broader, more encompassing explanatory scope of our proposed framework. From this perspective, our model achieves parsimony by explaining a wider array of empirical phenomena (i.e., the range-dependent biases observed in our experiments and relevant to interpreting past studies) through a single, coherent mechanism: the perception-action scaling factor α.

Furthermore, the notion that 360/360 studies are "preferable" potentially overlooks the diverse objectives and practical constraints of psychophysical research. Experimenters often utilize restricted response ranges for focused investigations or specific task requirements (e.g. learning of specific prior distributions). Recognizing and modeling potential response-scaling effects (on both bias and standard deviation) is therefore essential for the accurate interpretation of perceptual data gathered under such varied conditions. Our work explicitly addresses this perception-action transformation, providing crucial insights into how experimental design choices can systematically shape reported percepts.

In conclusion, we maintain that in combination with our experimental data, our extended model offers a valuable and necessary advancement by formally integrating a response-mapping stage whose influence is modulated by the experimental response range. It provides a more comprehensive account of heading estimation behavior, reconciling seemingly disparate findings and underscoring the critical interplay between perception and action in psychophysical reporting. Most importantly, it provides a quantitatively precisely testable hypothesis that provide clear directions for future empirical validation.
